# MicroRNA Profiling Revealed the Mechanism of Enhanced Cold Resistance by Grafting in Melon (*Cucumis melo* L.)

**DOI:** 10.3390/plants13071016

**Published:** 2024-04-02

**Authors:** Xinmei Lang, Xuan Zhao, Jiateng Zhao, Tiantian Ren, Lanchun Nie, Wensheng Zhao

**Affiliations:** 1 College of Horticulture, Hebei Agricultural University, Baoding 071000, China; 18230148260@139.com (X.L.); 18833246921@139.com (X.Z.); suyanm1c@163.com (J.Z.); rtt18295079086@163.com (T.R.); 2 Hebei Key Laboratory of Vegetable Germplasm Innovation and Utilization, Baoding 071000, China; 3 Ministry of Education of China-Hebei Province Joint Innovation Center for Efficient Green Vegetable Industry, Baoding 071000, China

**Keywords:** melon, low temperature, grafting, miRNAs, target genes

## Abstract

Grafting is widely used to improve the resistance to abiotic stresses in cucurbit plants, but the effect and molecular mechanism of grafting on cold stress are still unknown in melon. In this study, phenotypic characteristics, physiological indexes, small-RNA sequencing and expression analyses were performed on grafted plants with pumpkin rootstock (PG) and self-grafted plants (SG) to explore the mechanism of changed cold tolerance by grafting in melon. Compared with SG plants, the cold tolerance was obviously enhanced, the malondialdehyde (MDA) content was significantly decreased and the activities of antioxidant enzymes (superoxide dismutase, SOD; catalase, CAT; peroxidase, POD) were significantly increased in PG plants. Depend on differentially expressed miRNA (DEM) identification and expression pattern analyses, *cme-miR156b*, *cme-miR156f* and *chr07_30026* were thought to play a key role in enhancing low-temperature resistance resulting from grafting. Subsequently, 24, 37 and 17 target genes of *cme-miR156b*, *cme-miR156f* and *chr07_30026* were respectively predicted, and 21 target genes were co-regulated by *cme-miR156b* and *cme-miR156f*. Among these 57 unique target genes, the putative promoter of 13 target genes contained the low-temperature responsive (LTR) *cis*-acting element. The results of qRT-PCR indicated that six target genes (*MELO3C002370*, *MELO3C009217*, *MELO3C018972*, *MELO3C016713*, *MELO3C012858* and *MELO3C000732*) displayed the opposite expression pattern to their corresponding miRNAs. Furthermore, *MELO3C002370*, *MELO3C016713* and *MELO3C012858* were significantly downregulated in cold-resistant cultivars and upregulated in cold-sensitive varieties after cold stimulus, and they acted as the key negative regulators of low-temperature response in melon. This study revealed three key miRNAs and three putative target genes involved in the cold tolerance of melon and provided a molecular basis underlying how grafting improved the low-temperature resistance of melon plants.

## 1. Introduction

The melon (*Cucumis melo* L.) is an annual plant of the *Cucurbitaceae* family, and it is an important economic crop. Melons prefer warm climates and have a certain degree of resistance to heat but are very sensitive to cold [1]. Temperature is an important environmental factor affecting melon growth and the quality of fruit produced. The optimal growth temperature of melon is 25–35 °C/15–18 °C for the day/night periods [2,3]. The optimum temperature for melon also varies slightly between different varieties and changes slightly during the growth and development of the plants. However, melons will be subjected to severe cold stress below 15 °C, and the damage caused is irreversible when the temperature is below 6 °C for 5–6 days [4]. Therefore, low temperature is the main abiotic stress that limits the production and quality of melons in cool regions, such as northern and northeastern China, especially during the winter and the spring.

When the plants were subjected to abiotic stresses, abundant reactive oxygen species (ROS) were produced, which led to lipid peroxidation of cell membranes and promoted the generation of malondialdehyde (MDA). Excessive accumulation of MDA resulted in further damage to the cell membranes, and the content of MDA was an important indicator reflecting the damage degree of the cell membrane and plant resistance to abiotic stresses [5,6]. Low-temperature stress is known to induce high production of ROS and MDA, which damage the cell membrane and organelles [7,8]. In addition, low temperature also limits plant photosynthesis and respiration and leads to reduced growth or even death of melon plants [4,9].

Grafting acts as an important technique and is widely applied in horticultural crops. Grafting can maintain the excellent characteristics of the scion and effectively enhance resistance to multiple biotic and abiotic stresses in grafted plants relying on the adaptable roots of rootstocks [10,11,12,13]. Therefore, grafting is routinely practiced to improve the cold resistance of numerous vegetables, especially cucurbits [14,15,16]. After the application of grafting, the root vitality is increased, the cell membrane stability is enhanced, multiple antioxidant enzyme activities are induced and the impact of low temperature on photosynthesis is reduced, so the cold resistance is significantly improved in grafted plants [17]. For example, the relative electrolyte leakage and malondialdehyde content are significantly decreased in cucumber seedlings grafted onto luffa rootstocks compared with self-grafted plants [16]. In addition, the photochemical efficiency of cucumber plants grafted onto the gourd rootstock is significantly increased, which further leads to increased low-temperature tolerance in grafted seedlings [18]. In watermelon, the cold resistance of grafted plants is significantly enhanced by the application of pumpkin rootstock, and the lipid peroxidation and protein oxidation processes are restricted in grafted plants [16,19]. In melon, grafting also acts as an effective manner of improving the abiotic stresses such as heavy metal stress and salt stresses [20,21]. Grafting can change the contents of sugars and organic acid and regulate multiple enzyme activities such as sucrose phosphate synthase and POD in melon [20,22].

MicroRNA (miRNA) is a type of endogenous, non-coding, single-stranded small RNA with the length of 19 to 24 nt [23]. MiRNAs have been reported to widely exist in plants and participate in various growth and development processes such as seed germination, flower organ development, fruit enlargement and ripening [24,25,26,27,28]. Expectedly, miRNAs also play an important role in resisting abiotic stresses including drought, salt, high temperature or cold [29,30,31,32]. In *Arabidopsis*, miRNAs are first confirmed to respond to low temperature, and the cold tolerance is significantly improved in *Arabidopsis* plants upon the overexpression of *miR397*, *miR402* or *miR408* [33,34,35]. In rice, the constitutive expression of *miR319b* also results in enhanced low-temperature resistance [36]. Similarly, the overexpression of *sha-miR319d* increases the cold tolerance of tomato [37]. However, the cold tolerance of tomato plants is significantly decreased with the upregulation of *sly-miR156e-3p* or *miR164a* [38,39]. In apple, the survival rate of plants overexpressing *Mdm-miR160e* is less than that of wild-type plants under low-temperature stress of 0 °C for 8 h [40]. In turnip, *Bra-novel-miR3936-5p* is induced but *miR319e* and *miR166m-2* are suppressed by cold stress of −4 °C for 8 h [41]. These results suggest that the responses of miRNAs to low temperature are not conserved in different species, and miRNAs include positive and negative regulators of cold stress.

MiRNAs carry out their function by regulating the expression of target genes [42]. There is a negative regulatory relationship between miRNAs and their target genes, and miRNAs are able to cleave and degrade their target genes by a complete complementary combination manner [43,44]. For example, the overexpression of *OsmiR156* enhances the cold tolerance of rice by suppressing the transcripts of its target gene *OsSPL3* (*squamosa promoter-binding protein-like 3*) [25,45]. *Mdm-miR160* plays a negative role in freezing tolerance, but *MdARF17* is targeted by *Mdm-miR160* and acts as a positive regulator of freezing tolerance in apple [40]. Similarly, an *miR169* and *NF-YA/HAP2* module is proposed to participate in the low-temperature response of purple alfalfa by degradome sequencing analysis [46].

*CBF*s (C-repeat binding factors) are important regulators for the cold stress response of plants and belong to the *DREB* subfamily of *AP2/ERF* (APETALA2/ETHYLENE-responsive factor) family [47]. In *Arabidopsis*, the transcripts of *CBF* genes are rapidly induced after 15–30 min of cold treatment and peak at 1–3 h of cold stress [48]. The overexpression of *CBF3* increases the contents of proline and total soluble sugars and further improves the cold resistance of *Arabidopsis* [49]. The upregulation of *BnCBF5* and *BnCBF17* leads to depressed leaf electrolyte leakage and enhanced chilling tolerance compared with wild-type *Brassica napus* [50]. *CBFs* regulate cold tolerance through binding to C-repeat/dehydration responsive element (*CRT/DRE*, A/GCCGAC) *cis*-acting motifs in the promoters of cold-regulated genes (*CORs*) [51]. In sweet potato, *IbCBF3* induces the transcripts of *IbCOR27*, *IbCOR314* and *IbCOR413* by binding to *CRT/DRE* elements in the promoters of *COR* genes. The overexpression of *IbCBF3* enhances the cold stress tolerance of sweet potato and results in decreased contents of MDA and H_2_O_2_ under low-temperature stress [52]. In addition, cold response is also regulated by multiple transcription factors such as *WRKY*, *MYB*, *NAC* and the above-mentioned *SPLs* [53,54,55,56]. Ectopic expression of cucumber *CsWRKY46* into *Arabidopsis* leads to higher proline accumulation, less electrolyte leakage and lower MDA levels than that in the wild type, and the survival rate of seedlings is significantly improved in *CsWRKY46*-overexpressing plants after freezing treatment [57]. In apple, *MYB308L* positively regulates anthocyanin accumulation and enhances the low-temperature tolerance [58]. In pepper, *CaNAC2* is significantly induced by low temperature, and the inhibition of *CaNAC2* increases the sensitivity of pepper seedlings to low temperatures [59].

In melon production, grafting can effectively improve the resistance of plants to various abiotic stresses [20,21], but the effect and molecular mechanism of grafting on low-temperature stress are still not fully clear. MiRNAs and corresponding target genes actively participate in low-temperature stress responses [25,42]. However, the key miRNAs and target genes responding to low-temperature stress in melon are still poorly understood, and the regulatory networks between miRNAs and their target genes need further study. Therefore, phenotypic characteristics, physiological indexes, small-RNA sequencing and expression analyses were performed in SG and PG plants in this study to explore the relationship between grafting and low-temperature response and to investigate the mechanism underlying how the plants resist low temperatures in melon.

## 2. Results

### 2.1. Grafting with Pumpkin Rootstock Enhanced the Plant Resistance to Low Temperatures in Melon

**Figure 1:**
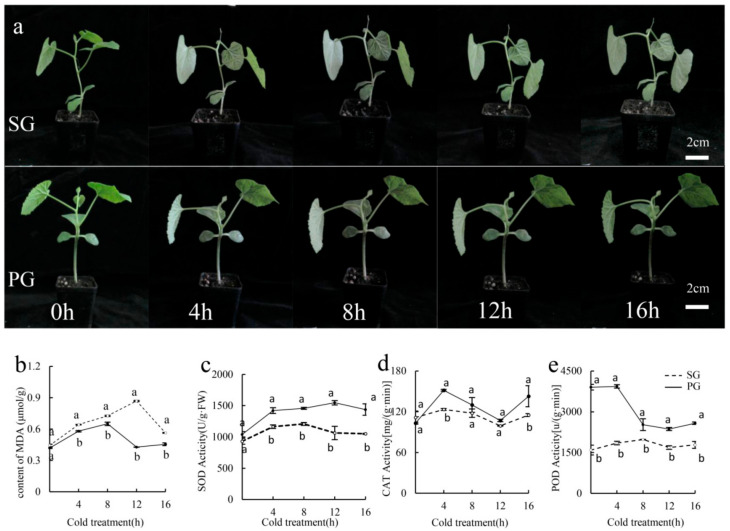
Morphological and physiological characteristics of SG and PG plants under low-temperature stress. (**a**) Phenotypic changes in PG and SG plants at 4 °C. (**b**) Content of MDA. (**c**–**e**) Antioxidase activities of SOD (**c**), CAT (**d**) and POD (**e**). Lowercase letters represent the significant differences in physiological indexes at *p* < 0.05 (Student’s *t*-test).

In melon production, grafting is an effective technique to enhance the plant resistance to multiple diseases and abiotic stresses [60]. In order to understand the effect of grafting on the low-temperature response of melon plants, morphological characteristics of self-grafted seedlings (SG) and seedlings grafted with pumpkin rootstock ‘YinGuang’ (PG) were investigated under low-temperature stress at 4 °C (Figure 1a). Both SG and PG plants dehydrated and wilted with the extension of low temperature, but the PG plants showed greater resistance to low temperature than SG plants (Figure 1a). After 12 h of low temperature, the phenotype differences were apparent, and self-grafted seedlings showed more severe wilting compared with PG plants (Figure 1a). Therefore, grafting with pumpkin rootstock effectively improved the resistance of melon plants to low-temperature stress.

Subsequently, the content of MDA and the activities of antioxidases (SOD, CAT and POD) were detected to understand the phenotype difference between SG and PG plants under low-temperature stress (Figure 1b–e). The MDA content in SG plants was distinctly induced by low temperature from 0 to 12 h but decreased at 16 h after cold treatment (Figure 1b). In PG plants, the MDA content was also significantly increased from 0 to 8 h but visibly declined at 12 h of low-temperature treatment (Figure 1b). In summary, the content of MDA in PG seedlings was always lower and decreased earlier than that in SG seedlings under low-temperature stress (Figure 1b). The activities of three antioxidant enzymes (SOD, CAT and POD) were significantly higher in PG seedlings than in SG seedlings at all detected time points after low-temperature treatment (Figure 1c–e). The variation tendency of SOD and CAT activities in SG seedlings was similar to that in PG seedlings (Figure 1c,d), but there was a difference in the initial POD content between SG and PG seedlings, which may result from the effect of grafting with pumpkin rootstock (Figure 1e). These results suggested that grafting with pumpkin rootstock could effectively decrease the MDA content and increase the activities of SOD, CAT and POD in melon seedlings under low-temperature stress.

### 2.2. Small-RNA Signatures of SG and PG Seedlings under Low-Temperature Stress

**Figure 2:**
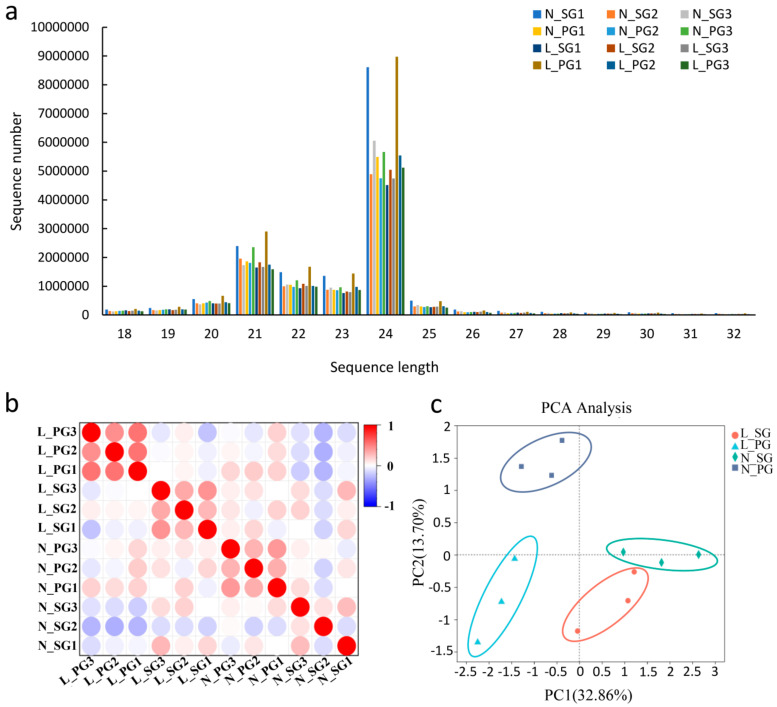
Small-RNA signatures of SG and PG seedlings under low-temperature stress. (**a**) Length distribution of sRNAs in twelve libraries. (**b**) Correlation between different samples. (**c**) Principal component analysis (PCA) of miRNA expression profiles.

MiRNAs have been reported to widely exist in various plants and play an important role in responding to low-temperature stress [24]. To investigate the effect of miRNAs on the resistance of grafted melon plants to low-temperature stress, small-RNA sequencing was performed on SG and PG seedlings treated with 4 °C for 12 h (L-SG/L-PG), and normal temperature (25 °C) was used for control (N-SG/N-PG). Three biological replicates were performed for each treatment, and thus 12 libraries were produced (Table S1). For each sample, 10.29 to 18.55 million raw reads were generated by high-throughput sequencing. After quality control of the original sequencing data, 9.97 to 18.14 million clean reads were obtained. According to the sRNA length characteristics, 18 nt to 32 nt (nucleotide) reads were determined as useful reads, and 9.33 to 17.27 million useful reads were obtained (Table S1). The length for most of small RNAs ranged from 20 nt to 25 nt, and the small RNAs with the length of 24 nt accounted for the largest proportion in all 12 libraries (Figure 2a). Finally, 7.00 to 13.01 million useful reads were mapped to the melon genome (http://cucurbitgenomics.org/organism/18 (accessed on 6 January 2023)) by Bowtie software (v1.2.3) (Table S1) [61].

The known miRNAs were acquired by miRBase and Rfam databases, and the novel miRNAs were identified by miRDeep2 software (v2.0.1.3) [62,63]. Meanwhile, repeated sequences and ncRNAs such as ribosomal RNA (rRNA), transport RNA (tRNA), small intracellular RNA (snRNA) and small nucleolar RNA (snoRNA) were filtered. In total, 105 to 112 known miRNAs and 104 to 106 novel miRNAs were identified for each sample (Table 1). After removing the repeated miRNAs from each library, 222 unique miRNAs were finally acquired and included 116 known miRNAs and 106 novel miRNAs (Table S2). The 116 known miRNAs belonged to 28 miRNA families in which the number of miRNAs in the miR156 (ten) and miR169 (twenty) families were the largest (Table S2).

The square of Pearson’s correlation coefficient (R^2^) between biological replicates in the same treatment was obviously higher than that in different treatments (Figure 2b). Principal component analysis (PCA) showed that samples from the same treatment were distinctly clustered together and clearly distinguished from samples of other treatments (Figure 2c). These results ensured the reliability of subsequent differentially expressed miRNA (DEM) identification, and suggested that the miRNAs profiles were different in N-SG, N-PG, L-SG and L-PG.

### 2.3. Identification of DEMs during Cold Response

In order to study the role of miRNAs in responding to cold stress, the expression levels of miRNAs were primarily investigated in SG and PG plants under low-temperature stress (L-SG vs. L-PG). Compared with L-SG, 16 miRNAs (*cme-miR156b*, *cme-miR156f*, *cme-miR169c*, *cme-miR169d*, *cme-miR169g*, *cme-miR169o*, *cme-miR169p*, *cme-miR169q*, *cme-miR169s*, *cme-miR530a*, *chr00_2081*, *chr00_3087*, *chr07_28660*, *chr07_30026*, *chr05_22190* and *chr09_33745*) were significantly increased, and 6 miRNAs (*cme-miR397*, *cme-miR398a*, *cme-miR398b*, *cme-miR399g*, *cme-miR408* and *chr08_32282*) were significantly decreased in L-PG (Table S3). However, the comparison between L-PG and L-SG included the effect of low temperature on miRNA expression and also contained the difference caused by grafting. Subsequently, the miRNA expression profiles of N-PG and N-SG were compared. In total, eight (*cme-miR169c*, *cme-miR169d*, *cme-miR169o*, *cme-miR169p*, *cme-miR169q*, *cme-miR169r*, *cme-miR169s* and *cme-miR169t*) and six (*cme-miR397*, *cme-miR398a*, *cme-miR399d*, *cme-miR399e*, *cme-miR399g* and *cme-miR408*) miRNAs were respectively upregulated and downregulated in N-PG compared with N-SG. The DEMs present in the comparison between L-PG and L-SG, but not N-PG and N-SG, were thought to be closely related to enhanced low-temperature resistance resulted from grafting in melon. A total of 12 DEMs were screened by this way and included *cme-miR156b*, *cme-miR156f*, *cme-miR169g*, *cme-miR398b*, *cme-miR530a*, *chr00_2081*, *chr00_3087*, *chr05_22190*, *chr07_28660*, *chr07_30026*, *chr08_32282* and *chr09_33745*.

In addition, the miRNA expression profiles of N-PG and L-PG were compared, and seven miRNAs were differentially expressed. Compared with N-PG plants, five miRNAs (*cme-miR156b*, *cme-miR156f*, *chr00_3087*, *chr07_30026* and *chr11_40459*) were significantly induced, whereas *cme-miR167c* and *chr00_2803* were significantly downregulated in L-PG plants (Table S3). Finally, the common DEMs (*cme-miR156b*, *cme-miR156f*, *chr00_3087* and *chr07_30026*) between the comparison set of N-PG vs. L-PG and the above-mentioned 12 DEMs were thought to play a key role in enhancing the low-temperature resistance of grafted plants with pumpkin rootstock.

**Figure 3:**
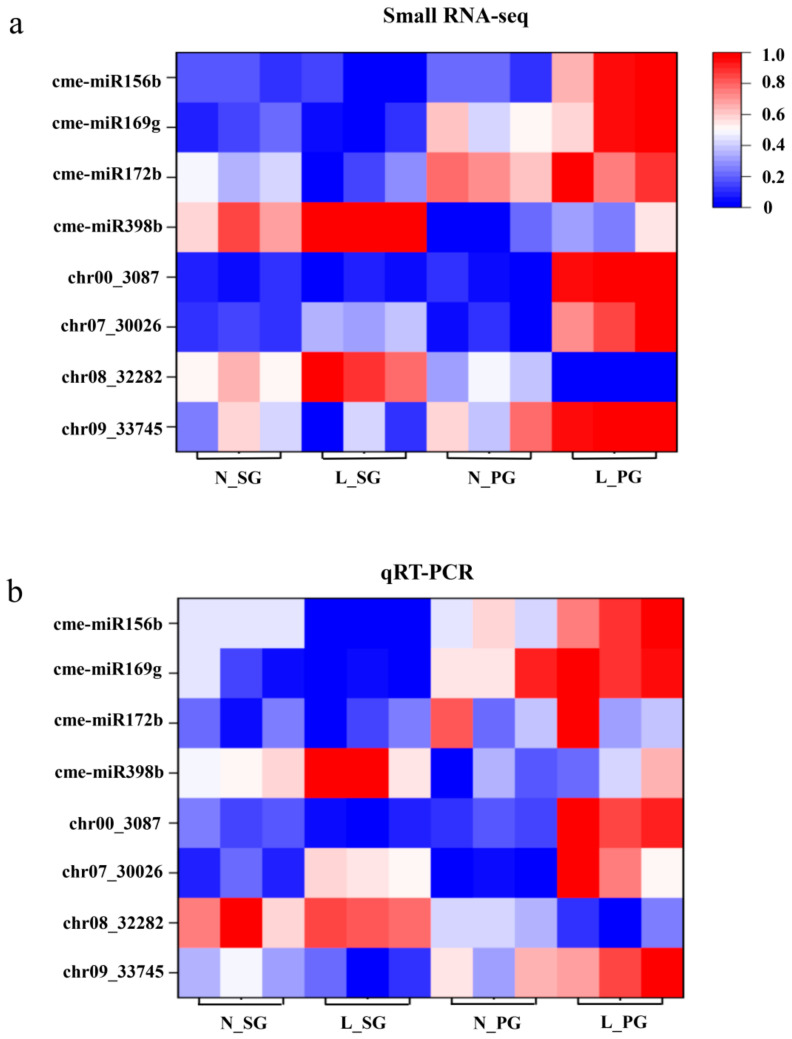
qRT-PCR confirmation of selected miRNAs. (**a**) The sRNA-seq data of selected DEMs. (**b**) The qRT-PCR data of selected DEMs. The Pearson’s correlation coefficient between sRNA-seq and qRT-PCR data was 0.82. Both sRNA-seq and qRT-PCR were performed at 12 h after cold (4 °C, L-SG/L-PG) or normal-temperature (25 °C, N-SG/N-PG) treatment.

In order to validate the DEM results, independent samples were collected in the same manner as in the DEM analysis, and eight DEMs were randomly chosen to perform the qRT-PCR analysis (Figure 3). The expression patterns of the selected DEMs detected by qRT-PCR were consistent with those from sRNA-seq data (Figure 3). The Pearson’s correlation coefficient between sRNA-seq and qRT-PCR data was 0.82, indicating highly reliable sRNA-seq results.

### 2.4. Expression Patterns of Four Key DEMs under Low-Temperature Stress and Target Gene Prediction

**Figure 4:**
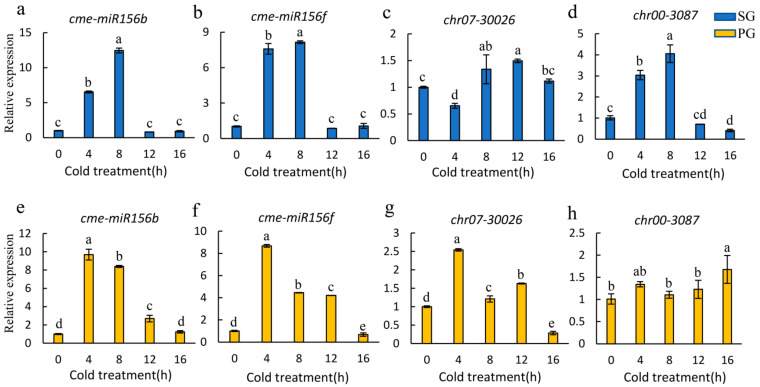
Expression patterns of four key miRNAs under low-temperature stress. The blue (**a**–**d**) and yellow (**e**–**h**) columns represent the expression levels of miRNAs in SG and PG plants, respectively. Lowercase letters above the columns represent significant differences at *p* < 0.05 (Duncan’s test).

In order to explore the response of four key DEMs to low-temperature stress, the expression levels of *cme-miR156b*, *cme-miR156f*, *chr00_3087* and *chr07_30026* were investigated in SG and PG seedlings under different low-temperature durations (Figure 4). In SG seedlings, the expression levels of *cme-miR156b*, *cme-miR156f* and *chr00_3087* were significantly increased at 4 h of cold treatment and reached the peak value at 8 h of low-temperature treatment but rapidly decreased after 12 h and then 16 h of low-temperature stress (Figure 4a,b,d). However, *chr07_30026* was significantly suppressed by cold treatment at 4 h and then exhibited increased expression compared with 0HAC (0 h after cold treatment) (Figure 4c). In PG seedlings, *cme-miR156b* and *cme-miR156f* were also significantly increased after low-temperature stress (4, 8 and 12 h) and returned to a similar level as 0HAC (Figure 4e,f). *Chr07_30026* was also significantly induced at 4 h of cold treatment and then declined with subsequent low-temperature treatment in PG seedlings (Figure 4g). However, there was no significant difference in the expression level of *chr00_3087* from 0 to 12 h of low-temperature stress, though slight upregulation was observed (Figure 4h). These results suggested that *cme-miR156b*, *cme-miR156f* and *chr07_30026* displayed an active response to low-temperature stress.

The functions of miRNAs were mainly dependent on post-transcriptional regulation of their target genes [64]. An miRNA was able to regulate multiple target genes, and one gene may also be regulated by different miRNAs [65]. In this study, 7556 and 5649 target genes were respectively predicted for 116 known miRNAs and 106 novel miRNAs by the online software TargetFinder (v5.8) (https://targetfinder.org/ (accessed on 13 November 2022)) (Table S4) [66]. In order to explore the regulators involved in low-temperature resistance mediated by grafting, the target genes of *cme-miR156b*, *cme-miR156f* and *chr07_30026* were identified. In total, 24 and 37 target genes were predicted for *cme-miR156b* and *cme-miR156f*, of which 21 target genes were co-regulated by *cme-miR156b* and *cme-miR156f* (Table S5). The number of target genes was 17 for the novel miRNA *chr07_30026* (Table S5). In total, 57 unique target genes regulated by *cme-miR156b*, *cme-miR156f* and *chr07_30026* were predicted (Table S5).

### 2.5. Expression Pattern Analyses of Key Target Genes under Low-Temperature Stress

**Figure 5:**
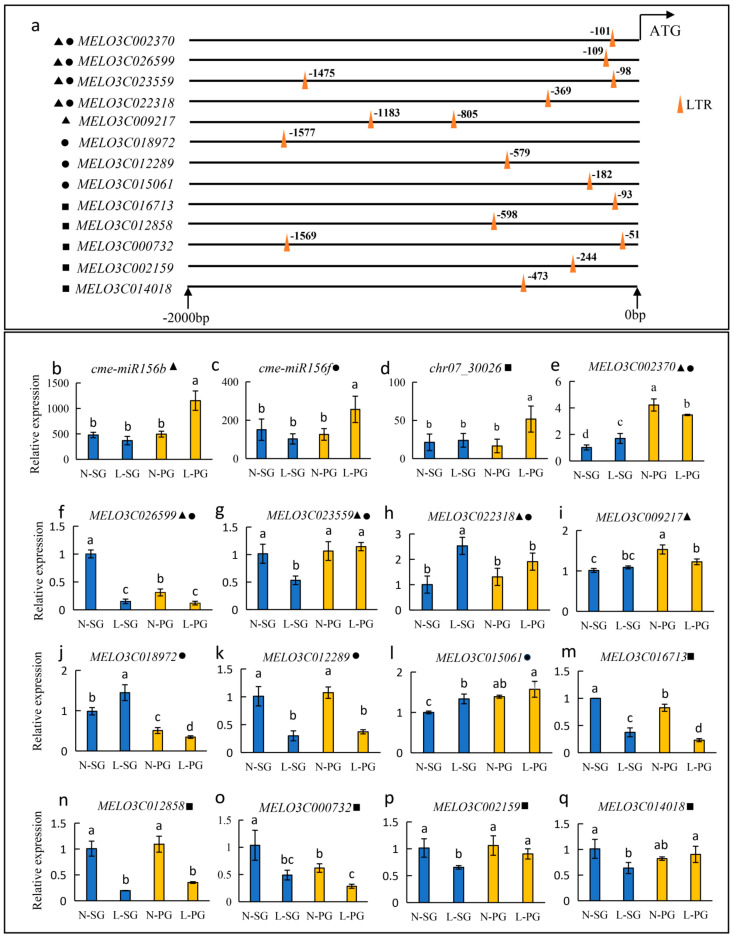
Comparison of expression patterns between key miRNAs and predicted target genes. (**a**) LTR (yellow triangles) *cis*-acting element analysis of target genes. (**b**–**q**) Expression analyses of three miRNAs (**b**–**d**) and their target genes (**e**–**q**) in SG (blue columns) and PG (yellow columns) plants after 12 h of normal or cold treatment. Black triangles, circles and rectangles represent the genes targeted by *cme-miR156b*, *cme-miR156f* and *chr07_30026*, respectively. Lowercase letters above the columns represent the significant differences at *p* < 0.05 (Duncan’s test).

The presence of LTR *cis*-acting elements plays an important role in regulating gene expression when plants encounter cold stimuli [67]. According to promoter sequence analysis, 13 out of 57 target genes possessed LTR *cis*-acting elements (Figure 5a), and the expression patterns of these 13 target genes were analyzed by qRT-PCR under low-temperature stress. In SG plants, the expression levels of *cme-miR156b* and *cme-miR156f* were slightly reduced, and the expression level of *chr07_30026* was slightly induced by low temperature in SG plants (Figure 5b–d). However, *cme-miR156b*, *cme-miR156f* and *chr07_30026* were significantly induced by cold in PG plants (Figure 5b–d). The putative target genes *MELO3C002370*, *MELO3C009217* and *MELO3C018972* exhibited the opposite expression patterns to *cme-miR156b* and *cme-miR156f* and were increased and decreased in L-SG and L-PG, respectively (Figure 5e,i,j). Similarly, *MELO3C016713*, *MELO3C012858* and *MELO3C000732* showed the inverse expression pattern to *chr07_30026* and were suppressed by low-temperature stress in SG and PG plants (Figure 5m–o). There were no opposite expression patterns between the other seven putative target genes (*MELO3C023559*, *MELO3C022318*, *MELO3C002159*, *MELO3C026599*, *MELO3C015061*, *MELO3C012289* and *MELO3C014018*) and relevant miRNAs (*cme-miR156b*, *cme-miR156f* and *chr07_30026*) (Figure 5). Therefore, *MELO3C002370*, *MELO3C009217*, *MELO3C018972*, *MELO3C016713*, *MELO3C012858* and *MELO3C000732* were considered the key candidate genes in regulating grafting-enhanced low-temperature resistance.

**Figure 6:**
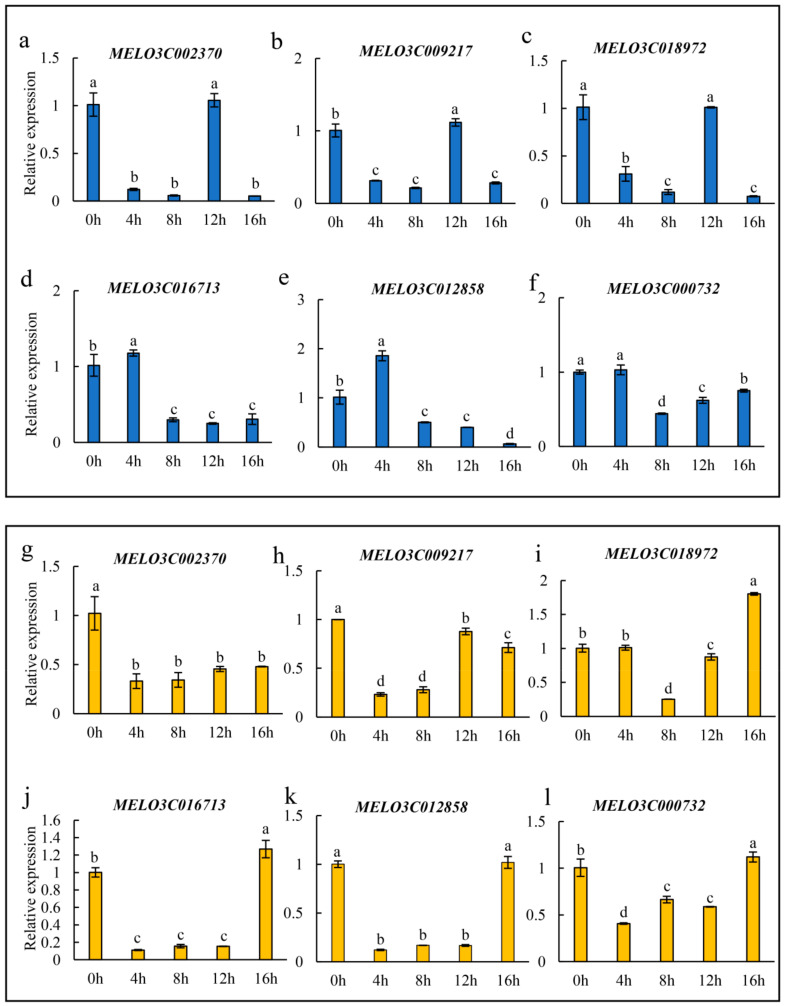
Expression patterns of predicted target genes under low-temperature stress. Expression patterns of target genes in SG (**a**–**f**, blue columns) and PG (**g**–**l**, yellow columns) plants after low-temperature treatment for 0 h, 4 h, 8 h, 12 h and 16 h. Lowercase letters above the columns represent the significant differences in transcript levels at *p* < 0.05 (Duncan’s test).

Subsequently, the expression levels of *MELO3C002370*, *MELO3C009217*, *MELO3C018972*, *MELO3C016713*, *MELO3C012858* and *MELO3C000732* were investigated in the dynamic low-temperature process of SG and PG plants (Figure 6a–l). In SG plants, *MELO3C002370*, *MELO3C009217* and *MELO3C018972* were significantly downregulated after 4 h of low temperature, but an unexpected upregulation was observed at 12 h of low temperature (Figure 6a–c). However, the downregulation of *MELO3C016713*, *MELO3C012858* and *MELO3C000732* was relatively delayed compared with that of *MELO3C002370*, *MELO3C009217* and *MELO3C018972* and significantly decreased after 8 h of low temperature (Figure 6d–f). In PG plants, all six above-mentioned candidate genes except for *MELO3C018972* were significantly suppressed from 4 h of low temperature (Figure 6g–l). The downregulation of *MELO3C016713*, *MELO3C012858* and *MELO3C000732* in PG plants occurred earlier than that in SG plants, and their expression levels were similar to the 0HAC until 16 h of low temperature (Figure 6j–l). The transcripts of *MELO3C009217* were significantly increased at 12 h and close to 0HAC at 16 h of low temperature (Figure 6h). It is worth noting that the expression level of *MELO3C002370* remained at a low level after 16 h of low-temperature treatment in PG plants (Figure 6g). *MELO3C018972* was significantly downregulated at 8 h of low temperature and showed a later downregulation in PG plants compared with that in SG plants (Figure 6c,i). Therefore, *MELO3C009217*, *MELO3C016713*, *MELO3C012858*, *MELO3C000732* and especially *MELO3C002370* may act as the major negative regulators in enhancing the cold resistance of PG plants.

**Figure 7:**
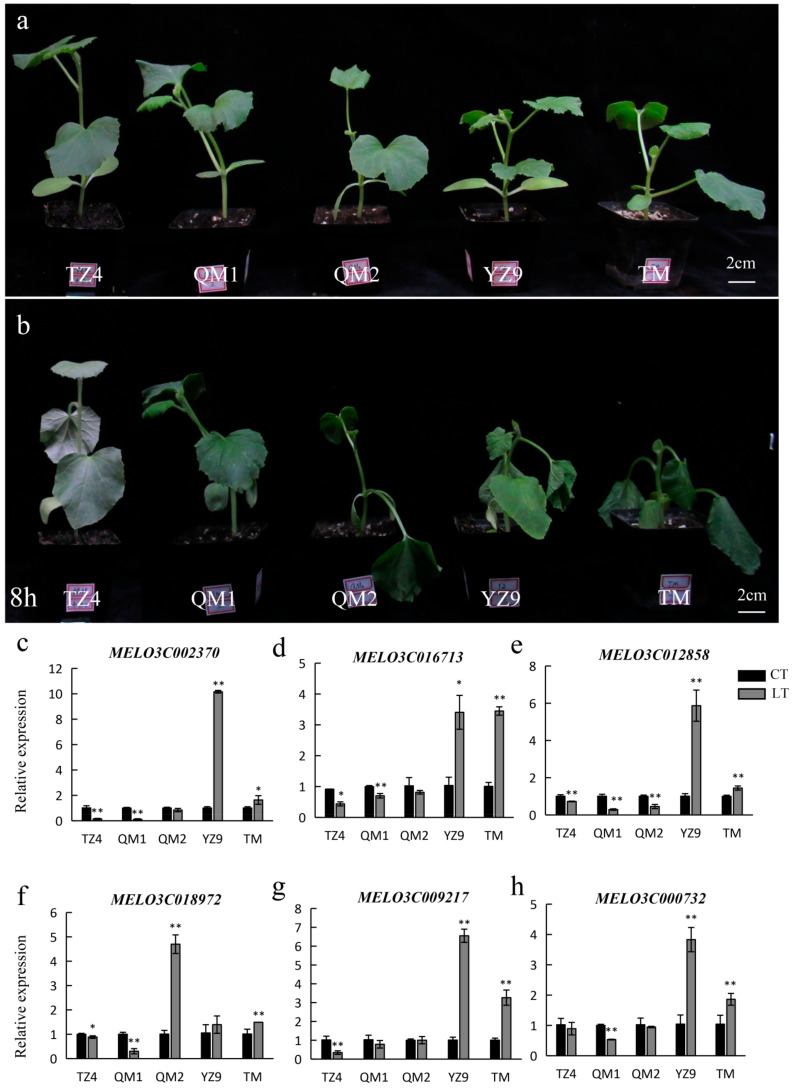
Expression analyses of key target genes in five cultivars of melon with different low-temperature resistance. (**a**,**b**) Phenotypic characteristics of five cultivars under low-temperature (4 °C) stress of 0 h (CK) (**a**) and 8 h (**b**). (**c**–**h**) Expression changes in six candidate target genes by low-temperature stress in cultivars of melon. CT and LT represent the normal (28 °C) and low-temperature (4 °C) treatments, respectively. Asterisks represent significant differences at the transcriptional level (Student’s *t*-test: *, *p* < 0.05; **, *p* < 0.01).

In addition, the expression levels of *MELO3C002370*, *MELO3C009217*, *MELO3C018972*, *MELO3C016713*, *MELO3C012858* and *MELO3C000732* were investigated in several cultivars of melon with different low-temperature resistance (strong, TZ4 and QM1; middle, QM2; weak, YZ9 and TM) (Figure 7a). Compared with control, the expression levels of *MELO3C002370*, *MELO3C016713* and *MELO3C012858* were significantly decreased in TZ4 and QM1 and significantly increased in YZ9 and TM under low-temperature stress (Figure 7b–d). In QM2, there was no significant difference in the transcripts of *MELO3C002370* and *MELO3C016713*, but *MELO3C012858* was significantly reduced after low-temperature stress (Figure 7b–d). The transcripts of *MELO3C009217*, *MELO3C018972* and *MELO3C000732* also changed in several melons, but their expression levels were not perfectly consistent with the low-temperature resistance of given cultivars (Figure 7e–g). These results further suggested that *MELO3C002370*, *MELO3C016713* and *MELO3C012858* were the key negative regulators of low-temperature response in melon. *MELO3C002370*, *MELO3C016713* and *MELO3C012858*, respectively, were the homologs of *SPL13*, ABA receptor *GCR2* and *RIC7* by BLAST searches in the Arabidopsis Information Resource (http://www.arabidopsis.org/ (accessed on 31 March 2005)) using their full-length amino acid sequences (Table 2).

## 3. Discussion

The stress resistance of plants is closely linked to the stability of the cell membrane and the accumulation of osmotic substances. When plants experience abiotic stresses, the permeability of the cell membrane increases, leading to the production of a large quantity of toxic substances such as MDA and ROS [7,8]. Fortunately, grafting can effectively reduce the accumulation of toxic substances, promote the production of osmotic substances such as proline and soluble protein, and enhance the antioxidant enzyme activity to improve the stress resistance of grafted plants [60,68]. At present, the effect and mechanism of grafting on cold tolerance are still not clear in melon. This study explored the differences in morphology and physiological indexes between PG and SG plants under low-temperature stress. Compared with SG plants, grafted seedlings with pumpkin rootstock showed enhanced cold tolerance, less MDA content and higher activities of SOD, CAT and POD (Figure 1). These results indicated that grafting with appropriate rootstock could effectively improve the resistance of melon plants to cold stress and significantly alter the levels of physiological indexes related to low-temperature response.

In order to investigate the mechanism of enhanced cold tolerance resulting from grafting, small-RNA sequencing was performed on SG and PG plants under low-temperature (L-SG/L-PG) and normal-temperature (N-SG/N-PG) conditions. Combined with DEM identification and expression pattern analyses, *cme-miR156b*, *cme-miR156f* and *chr07_30026* were identified as the major regulators of low-temperature response mediated by grafting. Previous studies have indicated that *miR156* is extensively involved in the response to cold stress [25,69]. In sugarcane, *miR156* is significantly induced by low-temperature stress, and the ectopic expression of sugarcane *miR156* leads to better growth status, lower ROS level, and higher anthocyanin content in tobacco leaves under cold conditions [70]. In rice, *OsmiR156* also positively regulates the cold stress tolerance by increasing cell viability and growth rate after low-temperature treatment [45]. However, *sly-miR156e-3p* of tomato acts as a negative regulator of cold tolerance, and the overexpression of *sly-miR156e-3p* displays more severe leaf wilting, lower maximal photochemical efficiency of PSII and higher relative electrolyte leakage compared with the wild type [38]. Therefore, the function of *miR156* is not conserved in different species. In this study, *cme-miR156b* and *cme-miR156f* were significantly induced by low-temperature stress in PG and SG plants (Figure 4a,b,e,f), which suggested that *cme-miR156b* and *cme-miR156f* acted as the positive regulators of cold stress in melon. At present, the functions of *cme-miR156b* and *cme-miR156f* in melon are still unknown, and their importance in responding to low-temperature stress remain to be clarified. In addition, a novel miRNA, *chr07_30026*, was also significantly increased after cold treatment in PG plants, though *chr07_30026* was suppressed by low temperature at 4 h in SG plants (Figure 4c,g).

MiRNAs mainly work by regulating the expression of target genes [71]. In tomato, *sha-miR319d* improves the cold tolerance of plants by inhibiting the transcription of *GAMYB-like 1* [37]. Similarly, *OsmiR528* inhibits the expression of *OsMYB30*, which negatively regulates *BMY4* (*β-amylase 4*), *BMY6* and *BMY10* to increase the activity of β-amylase and further improves the low-temperature tolerance of rice [72]. In this study, 24, 37 and 17 target genes of *cme-miR156b*, *cme-miR156f* and *chr07_30026* were respectively predicted, and 13 target genes with the LTR *cis*-acting element were chosen to further analyze (Figure 5a). In total, six target genes (*MELO3C002370*, *MELO3C009217*, *MELO3C018972*, *MELO3C016713*, *MELO3C012858* and *MELO3C000732*) showed the opposite expression pattern to their corresponding miRNAs (Figure 5b–q). In addition, the expression patterns of the above-mentioned six target genes under low-temperature stress were investigated in several cultivars of melon with different low-temperature resistance, and three target genes (*MELO3C002370*, *MELO3C016713* and *MELO3C012858*) were downregulated in cold-resistant cultivars and upregulated in cold-sensitive varieties (Figure 7). These results suggested that *MELO3C002370*, *MELO3C016713* and *MELO3C012858* acted as the key negative regulators of cold stress response mediated by grafting in melon.

The results of homologous alignment using the full-length amino acid sequence showed that *MELO3C002370*, *MELO3C016713* and *MELO3C012858* were the homologs of *Arabidopsis SPL13*, ABA receptor *GCR2* and *RIC7*, respectively (Table 2). However, the functions of putative *CmSPL13* (*MELO3C002370*), *CmGCR2* (*MELO3C016713*) and *CmRIC7* (*MELO3C012858*) are still not clear in melon. In *Arabidopsis*, *SPL13* promotes floral transition and regulates adult leaf morphology by directly repressing *BLADE-ON-PETIOLE 1* (*BOP1*) and *BOP2* [73]. In addition, the homologs of *SPL13* are also involved in counteracting drought, heat and flooding stresses in *Medicago sativa* [74,75,76]. At present, the function of *SPL13* in cold stress response is still unknown. *GCR2* functions as a G-protein-coupled receptor (*GPCR*) for ABA, but no obvious morphological defect is observed in loss-of-function alleles of *Arabidopsis GCR2*, which may result from the subtle phenotypes of *GCR2*, the functional redundancy with other genes or the exhibition of phenotypes only under certain conditions [77,78]. *RIC7* has been found to negatively regulate ABA-induced stomatal closure. Compared with the wild type, plants with overexpression of *RIC7* exhibit larger stomatal apertures, accumulate less H_2_O_2_ and reduce the expression levels of genes related to ROS generation after ABA treatment [79].

Melons prefer warm climates and have a certain degree of resistance to heat but are very sensitive to cold. It is urgent to breed a melon variety with strong cold tolerance, but the molecular mechanism for the low-temperature response of melon is still unclear. According to the existing research and the results of this study, *cme-miR156b*, *cme-miR156f* and their common target gene *CmSPL13* were considered the key regulators for low-temperature response during grafting in melon. In the future, the function and mechanism of the *cme-miR156b*/*cme-miR156f*-*CmSPL13* module in regulating cold tolerance will be explored to further understand the interaction between melon plants and the environment. The results of this study provided a theoretical basis for breeding new melon varieties with high cold tolerance, and it is of great significance for the high-quality and efficient production of melon.

## 4. Materials and Methods

### 4.1. Plant Materials and Treatments

The melon variety ‘Boyang61’ was used as the scion, and ‘Yin Guang’ (*Cucurbita maxima* Duch.) was used as the rootstock. The controls were self-grafted plants of ‘Boyang61’ (one plant of ‘Boyang61’ was used as the scion and grafted onto another ‘Boyang61’ rootstock). When the seedlings of rootstocks reached the one-true-leaf stage and the cotyledons of scions displayed, grafting was performed using the cut-grafting method [80]. The grafted seedlings were grown in the greenhouse of Hebei Agricultural University under 25–30 °C and 85–100% relative humidity for approximately 7 days [81]. After the complete healing of grafted seedlings, the plants were transferred to standard management practices. At the 4-true-leaves stage (about 35 days after grafting), the apex of grafted plants was collected at 0 h, 4 h, 8 h, 12 h and 16 h after cold treatment (4 °C). The samples were rapidly frozen by liquid nitrogen and stored at −80 °C for physiological indicator determination and small-RNA-seq library construction.

### 4.2. Determination of Physiological Indicators

Malondialdehyde (MDA) was estimated from the third true leaves of PG and SG plants using 5% trichloroacetic acid (TCA) according to previous methods [8]. A 2 mL aliquot of enzyme extract was mixed with 10% TCA containing 0.65% (*w*/*v*) thiobarbituric acid (TBA), placed in a water bath at 100 °C for 30 min and then immediately cooled to stop the reaction. The reaction mixture was centrifuged for 10 min at 800× *g*, and the optical density (OD) value was measured at 532 nm, 600 nm and 450 nm.

For superoxide dismutase (SOD) determination, 20 μL enzyme extract was mixed with 3 mL SOD reaction mixture (50 mM phosphate buffer (pH 7.8), 0.75 mM nitro blue tetrazolium (NBT), 130 mM methionine, 0.02 mM riboflavin and 0.1 mM EDTA Na_2_). For peroxidase (POD), 0.1 mL enzyme extract was mixed with 2.9 mL POD reaction mixture (0.05 mM phosphate buffer (pH 6.0), 0.05 mM guaiacol and 2% (*w*/*v*) H_2_O_2_). For catalase (CAT), 0.1 mL enzyme extract was mixed with 3 mL reaction mixture (0.1 mM phosphate buffer (pH 7.0), 0.1 mM H_2_O_2_). The absorbance of SOD, POD and CAT was measured at 560 nm, 470 nm and 240 nm, respectively [82].

### 4.3. Small-RNA Library Construction and Small-RNA Sequencing

Total RNA was isolated from the leaves of PG and SG plants after low-temperature treatment for 0 h (control) and 12 h and checked on 1% agarose gels to avoid possible degradation and contamination. A NanoDrop 2000 photometer spectrophotometer and Agilent 2100 Nano were used to determine the RNA concentration and integrity. A 3 μg portion of total RNA for each sample was used for library construction by Majorbio Bio-Pharm Technology (Shanghai, China) using the Illumina TruSeq Small RNA Kit on an Illumina Novaseq XPlus platform.

### 4.4. Identification of Known and Novel miRNAs

After Illumina sequencing, the online software Fastx-Toolkit (v0.0.14) (http://hannonlab.cshl.edu/fastx_toolkit/ (accessed on 5 January 2014)) was used to filter raw reads, and the clean reads with a length of 18 nt to 32 nt were determined as useful reads that were mapped to the Melon (DHL92) v3.6.1 Genome (http://cucurbitgenomics.org/organism/18 (accessed on 6 January 2023)) by Bowtie (http://bowtie-bio.sourceforge.net/index.shtml (accessed on 6 July 2019)). The mapped reads were used to predict the known miRNAs by blasting in the miRBase 22.1 (http://www.mirbase.org/ (accessed on 7 October 2018)) database. The Rfam database (http://rfam.xfam.org/ (accessed on 30 May 2022)) was used to annotate ncRNAs and repeated sequences such as ribosomal RNA (rRNA), transport RNA (tRNA), small intracellular RNA (snRNA) and small nucleolar RNA (snoRNA). Furthermore, the unannotated reads containing potential miRNAs were obtained and mapped to the Melon (DHL92) v3.6.1 Genome. Combined with the surrounding sequences, the secondary structure was predicted by miRDeep2 (https://www.mdc-berlin.de/content/mirdeep2-documentation (accessed on 11 November 2019)). Subsequently, the novel miRNAs were identified according to some symbolic characteristics such as dicer enzyme cleavage site and energy value.

Transcripts per million (TPM) reads were used to evaluate the relative expression levels of each miRNA, and DESeq2 software (v2.0.1.3) was used to identify the DEMs [83]. The Bonferroni method was used to adjust the observed significance level. The miRNAs with at least a 2-fold change in expression and a *p*-adjusted of less than 0.05 were considered differentially expressed.

### 4.5. Identification of LTR Cis-Acting Elements

The 5′-upstream 2 kb region (putative promoter) of 57 target genes (Table S5) were obtained from the melon genome (http://cucurbitgenomics.org/organism/18, (accessed on 6 January 2023), v3.6.1), and used for LTR *cis*-acting element identification. The online software PlantCARE (v1.0) (https://bioinformatics.psb.ugent.be/webtools/plantcare/html/ (accessed on 7 September 1998)) was used to scan the sequences CCGAAA for LTR *cis*-acting elements [84].

### 4.6. Quantitative Real-Time RT-PCR (qRT-PCR)

The leaves of PG and SG plants treated by low temperature for 0 h, 4 h, 8 h, 12 h and 16 h were used for qRT-PCR experiments. The expression levels of *MELO3C002370*, *MELO3C009217*, *MELO3C018972*, *MELO3C016713*, *MELO3C012858* and *MELO3C000732* were investigated in melon cultivars (TZ4, QM1, QM2, YZ9 and TM) at 8 h of normal (28 °C) and low-temperature (4 °C) treatment. For miRNA expression analyses, the total RNA was extracted by the miRcute miRNA extraction and separation kit (TIANGEN, Beijing, China), the first strand cDNA was synthesized by the miRcute Plus miRNA first strand cDNA kit (TIANGEN, Beijing, China) and the expression levels were detected by the miRcute Plus miRNA qPCR kit (TIANGEN, Beijing, China). For target gene expression analyses, the total RNA was extracted by the RNA Easy Fast Plant Tissue kit (TIANGEN, Beijing, China), the first strand cDNA was synthesized by the Hifiscript gDNA removable RT master mix (CWBIO, Taizhou, China) and the expression levels were detected by the 2 × SYBR Green qPCR Master Mix (UE EVERBRIGHT, Sayreville, NJ, USA). A CFX96 TouchTM qRT PCR assay system (BioRad, Hercules, CA, USA) was used for qRT-PCR experiments, and each miRNA or gene was analyzed with three biological and three technical replicates. *SnRNA U6* and *CmActin* (*MELO3C008032*) of melon were used as the internal control. The relative expression levels of miRNAs and target genes were calculated by the 2^−∆∆ct^ method [85]. Specific primers of miRNAs and target genes were designed by Primer Premier 5 and listed in Table S6.

## 5. Conclusions

In this study, phenotypic characteristics, physiological indexes, small-RNA sequencing and expression analyses were performed in SG and PG plants to explore the mechanism of changed low-temperature resistance by grafting in melon. Grafting with pumpkin rootstock could effectively improve the resistance of melon plants to low-temperature stress, certified by alleviated wilting, decreased MDA content, and increased SOD, CAT and POD activities. The results of small-RNA sequencing and qRT-PCR suggested that *cme-miR156b*, *cme-miR156f* and *chr07-30026* acted as the positive regulators in resisting low-temperature stress and were significantly induced by cold in PG plants. Combined with target prediction and expression analyses, *CmSPL13* (*MELO3C002370*), *CmGCR2* (*MELO3C016713*) and *CmRIC7* (*MELO3C012858*) were thought to play a negative role in the cold resistance of melon plants. These results provided a basic regulatory network between the key miRNAs and their target genes involved in cold tolerance and enriched the mechanism underlying how grafting improved the low-temperature resistance of melon plants.

## Figures and Tables

**Table 1:** Numbers of known and novel miRNAs in 12 libraries.

	N-SG1	N-SG2	N-SG3	L-SG1	L-SG2	L-SG3	N-PG1	N-PG2	N-PG3	L-PG1	L-PG2	L-PG3
Known miRNAs	109	110	105	111	108	108	109	111	111	112	109	107
Novel miRNAs	106	105	104	105	104	105	106	104	105	105	105	105

**Table 2:** Information for MELO3C002370, MELO3C016713 and MELO3C012858.

ID	Rename	Localization	CDS Length (bp)	Best Hits to Arabidopsis	Functional Description in Arabidopsis
MELO3C002370	CmSPL13	chr12: 24537602–24540376 (+)	969	AT5G50570/AtSPL13	Involved in floral transition
MELO3C016713	CmGCR2	chr07: 2653267–2655968 (−)	1239	AT1G52920/AtGCR2	Encodes a plasma membrane-localized ABA receptor, which interacts with the Gαβγ complex
MELO3C012858	CmRIC7	chr04: 13342065–13344382 (+)	636	AT4G28556/AtRIC7	Encodes RIC7, the downstream effector of active Rop2 GTPase

## Data Availability

All the experimental data are contained within this article or the Supplementary Materials. The small-RNA sequencing data for SG and PG plants are available in the Sequence Read Archive (SRA) database at the National Center for Biotechnology Information (https://www.ncbi.nlm.nih.gov/ (accessed on 4 November 1988)) under accession number PRJNA1090144.

## Supplementary Materials

The following supporting information can be downloaded at: https://www.mdpi.com/article/10.3390/plants13071016/s1, Table S1: Summary of small-RNA sequencing data. Table S2: Information on identified miRNAs. Table S3: Differentially expressed miRNAs in this study. Table S4: Predicted targets for 116 known miRNAs and 106 novel miRNAs. Table S5: Target genes of *cme-miR156b*, *cme-miR156f* and *chr07_30026*. Table S6: Primer information in this study.
