# Supplementary material for: MicroRNA Profiling Revealed the Mechanism of Enhanced Cold Resistance by Grafting in Melon (Cucumis melo L.)
Source: Plants (Basel). 2024 Apr 2;13(7):1016. doi: 10.3390/plants13071016 (PMC11013280; doi:10.3390/plants13071016)
Supplement: Supplementary file 1 [file plants-13-01016-s001.zip › Supplementary Files/Table S5-Target genes of cme-miR156b, cme-miR156f and chr07_30026.docx]

**Table S5 Target genes of *cme-miR156b*, *cme-miR156f* and *chr07_30026***

| **miRNA name** | **Target gene numbers** | **Target gene ID** | **Target gene description** |
| --- | --- | --- | --- |
| *cme-miR156b/f* | 21 | ***MELO3C002370*** | **squamosa promoter-binding-like protein 13A** |
|  |  | ***MELO3C026599*** | **transcription factor bHLH106-like** |
|  |  | ***MELO3C023559*** | **40S ribosomal protein S27** |
|  |  | ***MELO3C022318*** | **squamosa promoter-binding-like protein 16** |
|  |  | *MELO3C002618* | Squamosa promoter-binding-like protein |
|  |  | *MELO3C025597* | Squamosa promoter-binding protein |
|  |  | *MELO3C009639* | Squamosa promoter binding protein |
|  |  | *MELO3C017245* | squamosa promoter-binding-like protein 6 |
|  |  | *MELO3C013813* | Cystic fibrosis transmembrane conductance regulator |
|  |  | *MELO3C016149* | Cationic amino acid transporter, putative |
|  |  | *MELO3C002048* | squamosa promoter-binding-like protein 12 isoform X1 |
|  |  | *MELO3C013006* | OXS3 |
|  |  | *MELO3C005752* | Short-chain dehydrogenase TIC 32, chloroplastic |
|  |  | *MELO3C003968* | Transcription factor |
|  |  | *MELO3C005966* | Squamosa promoter-binding-like protein |
|  |  | *MELO3C014895* | squamosa promoter-binding-like protein 13A |
|  |  | *MELO3C026196* | squamosa promoter-binding-like protein 13A |
|  |  | *MELO3C009391* | Glycosyltransferase |
|  |  | *MELO3C026536* | Starch branching enzyme |
|  |  | *MELO3C009823* | Proteasome subunit beta type-4 |
|  |  | *MELO3C034429* | Unknown protein |
| *cme-miR156b* | 3 | ***MELO3C009217*** | **Plant/protein** |
|  |  | *MELO3C007083* | 187-kDa microtubule-associated protein AIR9 |
|  |  | *MELO3C011078* | GATA transcription factor, putative |
| *cme-miR156f* | 16 | ***MELO3C018972*** | **Arabinogalactan-protein** |
|  |  | ***MELO3C012289*** | **Cyclin-D1-binding protein 1 like** |
|  |  | ***MELO3C015061*** | **Growth inhibition and differentiation-related protein 88** |
|  |  | *MELO3C003619* | serine/arginine-rich splicing factor RSZ22A |
|  |  | *MELO3C024020* | 60S ribosomal protein L44 |
|  |  | *MELO3C019024* | At5g64090 |
|  |  | *MELO3C003751* | ER lumen protein retaining receptor |
|  |  | *MELO3C035105* | Unknown protein |
|  |  | *MELO3C029072* | Unknown protein |
|  |  | *MELO3C024066* | Unknown protein |
|  |  | *MELO3C021481* | Overexpressor of cationic peroxidase 3, putative |
|  |  | *MELO3C003428* | ABC transporter B family-like protein |
|  |  | *MELO3C006539* | photosystem I reaction center subunit II, chloroplastic |
|  |  | *MELO3C031948* | Unknown protein |
|  |  | *MELO3C034464* | Unknown protein |
|  |  | *MELO3C031687* | Unknown protein |
| *chr07_30026* | 17 | ***MELO3C016713*** | **LanC-like protein GCR2** |
|  |  | ***MELO3C012858*** | **CRIB domain-containing protein RIC7** |
|  |  | ***MELO3C000732*** | **Copper centre Cu(A)** |
|  |  | ***MELO3C002159*** | **Nascent polypeptide-associated complex subunit beta** |
|  |  | ***MELO3C014018*** | **Pentatricopeptide repeat-containing protein family** |
|  |  | *MELO3C035115* | Unknown protein |
|  |  | *MELO3C034147* | histone-lysine N-methyltransferase ASHR1 isoform X1 |
|  |  | *MELO3C034146* | histone-lysine N-methyltransferase ASHR1 isoform X1 |
|  |  | *MELO3C009296* | Energy-coupling factor transporter ATP-binding EcfA 1 |
|  |  | *MELO3C024737* | Unknown protein |
|  |  | *MELO3C027561* | Unknown protein |
|  |  | *MELO3C010862* | Hexosyltransferase |
|  |  | *MELO3C028914* | histone-lysine N-methyltransferase ASHR1 isoform X1 |
|  |  | *MELO3C034106* | Unknown protein |
|  |  | *MELO3C013648* | Unknown protein |
|  |  | *MELO3C026135* | Unknown protein |
|  |  | *MELO3C025218* | Unknown protein |

The gene ID shown in bold indicates that their promoter region contains LTR *cis*-acting elements.
