# Supplementary material for: MicroRNA Profiling Revealed the Mechanism of Enhanced Cold Resistance by Grafting in Melon (Cucumis melo L.)
Source: Plants (Basel). 2024 Apr 2;13(7):1016. doi: 10.3390/plants13071016 (PMC11013280; doi:10.3390/plants13071016)
Supplement: Supplementary file 1 [file plants-13-01016-s001.zip › Supplementary Files/Table S6-Primer information in this study.docx]

Primer Sequence of miRNAs

| **MiRNA** | **Forward primer(5’-3’)** |
| --- | --- |
| cme-miR156b | GCCTTGACAGAAGATAGAGAGCAC |
| cme-miR156f | GCCGCTGACAGAAGATAGAGAGCAC |
| cme-miR169g | GCCGAAGCCAAGGATGAATTGCCGG |
| cme-miR398b | CCGCTGTGTTCTCAGGTCACCCCTT |
| cme-miR172b | CGCCGAGAATCTTGATGATGCTGCAT |
| chr08_32282 | CGCTGCCAAAAGAGACTTGCCCTG |
| chr09_33745 | CCCGCCATGTTATTGAGGAAGCTCTTG |
| chr00_3087 | AGCGCCTGTTTAGATTGAAGAAGT |
| chr07_30026 | GCGATTTATCATTGGTAGACTTTGC |
| U6(F) | CCCTTCGGGGACATCCGATA |
| U6(R) | CCATTTCTCGATTTGTGCGTGT |

Primer Sequence of Target Genes

| **Target Genes ID** | **Forward primer(5’-3’)** | **Reverse primer(5’-3’)** |
| --- | --- | --- |
| MELO3C000732.2 | ATGACTCAAGGTGAATCCCAC | TCAATCTACACAGAGTGAATCGG |
| MELO3C018972.2 | TCCTCCATCACCACCACCAGAAG | CCACCATCATTCCTCGGCTAACG |
| MELO3C016713.2 | TCCGCCACTCTCATTCCGACAG | GATGCCTTCCACTCAACGTCCAC |
| MELO3C012858.2 | GGCGGAAGAAATCAAAGGAGGAAGG | CAAGTCATCGTCGTTGGATCTGGAG |
| MELO3C009217.2 | CGGTGGTAAATCACATGCTGTTTCG | TCTGCTTGATGTGCTCCCATTCTTG |
| MELO3C002370.2 | GAGGCGGCAGCGAAAGGAAC | GCTGTTGTTGTTGTGGCTGATGATC |
| MELO3C023559.2 | TCATTTTGGTTGAGGAAGAGG | TCCAGATGACTGTCTTGTTATGG |
| MELO3C012289.2 | GGTGCTGGTCCTACTTTGTCTTCTG | ACTGCGGAACGACTTGGTTTGG |
| MELO3C014018.2 | TGGATGTTCTCTGTTCCTCGT | AGTAAGCAACGAAAACGAAGAG |
| MELO3C002159.2 | ATGGATCATACAAAGCTCCAGA | CTAAGAGGCTTGCTTCTCTTCT |
| MELO3C026599.2 | AAGTGGTTGAGCGAGTGAAAGAGC | CACAAGCCTTCCATCCTCTGACTTC |
| MELO3C022318.2 | GTTCTTCGACTCTGACTGTGCTCTC | CAAGACCGTCGCTGTGAATGAAATG |
| MELO3C015061.2 | TGGAGCGAGACTGTGGAAGACC | AGAGCGGACAAGTCGGTGAGAG |
| CmActin/MELO3C008032 | ATTCTTGCATCTCTAAGTACCTTCC | CCAACTAAAGGGAAATAACTCACC |
